# Psychometric properties of the dementia knowledge assessment scale-traditional Chinese among home care workers in Taiwan

**DOI:** 10.1186/s12888-021-03530-6

**Published:** 2021-10-19

**Authors:** Huei-Chuan Sung, Hsin-Feng Su, Hsiu-Mei Wang, Malcolm Koo, Raymond Y. Lo

**Affiliations:** 1 Graduate Institute of Long-term Care, Tzu Chi University of Science and Technology, No. 880, Sec. 2, Chien-Kuo Road, Hualien, 970046 Taiwan; 2 Institute of Medical Sciences, Tzu Chi University, Hualien, Taiwan; 3 Department of Nursing, Tzu Chi University of Science and Technology, Hualien, Taiwan; 4 Division of Cognitive/Geriatric Neurology, Department of Neurology, Hualien Tzu Chi Hospital, Hualien, Taiwan; 5 School of Medicine, Tzu Chi University, Hualien, Taiwan

**Keywords:** Dementia knowledge, DKAS, Home care worker, Confirmatory factor analysis, Psychometric, Traditional Chinese

## Abstract

**Background:**

The Dementia Knowledge Assessment Scale (DKAS) is a reliable and valid measurement of dementia knowledge for diverse allied health professionals but its traditional Chinese version has not been formally validated yet. The purpose of this study was to translate the DKAS from English to traditional Chinese and evaluate its psychometric properties among home care workers in Taiwan.

**Methods:**

The DKAS scale was translated into traditional Chinese through a forward translation and back translation process following the cross-cultural translation guideline. A total of 285 home care workers in eastern Taiwan were recruited using convenience sample. A total of 252 participants completed the questionnaires, giving a response rate of 88.4%. We tested the construct validity by confirmatory factor analysis (CFA) and evaluated the reliability by internal consistency.

**Results:**

The results of the CFA supported the 25-item, four-factor model for the DKAS-TC. The DKAS-TC achieved a good overall Cronbach’s alpha of .93 and McDonald’s omega of 0.94 with acceptable subscales McDonald’s omega ranged from .77 to .82.

**Conclusions:**

The DKAS-TC has adequate construct validity and reliability and can serve as an assessment tool to evaluate the knowledge level of home care workers in a dementia training program in Taiwan. The dementia knowledge level among home care workers in Taiwan was inadequate. There is a need for developing suitable dementia care training tailored to their learning needs and educational levels, and to improve their quality of care for those with dementia.

## Introduction

Due to the global population aging, the number of people with dementia is increasing rapidly. There are over 50 million people living with dementia currently worldwide and the number is estimated to reach 152 million by 2050 [1]. Taiwan is in a transitional phase from an aged society to a super-aged society due to a decrease in birth rate and a fast-aging population by 2025 [2]. The number of people diagnosed with dementia has also increased rapidly. About 1 in every 77 persons is diagnosed with dementia, and this number is projected to rise to more than double by 2040. The prevalence of dementia among people age 65 and older has reached 7.71% (over 300,000 people) in 2020 [3].

Dementia is a debilitating syndrome of brain disorders, which cause gradual deterioration in memory, thinking, judgement, behavior and the ability to perform everyday activities, and one of the leading causes of disability and dependency among older people. It can have negative impacts not only on the individuals with dementia, but also on their families and caregivers [4]. People with dementia often display behavioral and psychological symptoms which was reported by Taiwanese home care workers as the most challenging care problems [5]. More than 90% of older people with dementia live in the community, and are cared by family members in Taiwan. It was reported that 30.7% of families with relatives with dementia employed a foreign caregiver; but only 4.8% used home care services and 0.2% used day care services [6]. Some of the reasons for under-diagnosis and low utilization of long-term care services might be due to public stigma related to dementia, underreport by patients and families, and families’ unawareness of services. Most families might treat memory or cognitive decline as a part of normal aging process [7]. A recent study found that stigma towards dementia is prevalent and deeply rooted in Taiwan general public and reported as the major barrier to seek diagnostic examinations and for caregivers to utilize services and ask for support [8]. Su et al. also found that Taiwanese home care workers only had moderate level of knowledge of dementia and lacked dementia care training [5]. Lack of knowledge of dementia and stigma towards people with dementia may delay early diagnosis, medical treatment, and service referral [9]. With the modest level of the knowledge about dementia [5, 8] was reported in Taiwan, researchers suggest that the public should be educated on knowledge and friendly attitudes towards dementia, as well as students and professions of allied health [8].

To understand how well people know about dementia, several scales have been developed to measure dementia knowledge, such as the Alzheimer’s Disease Knowledge Scale (ADKS) [10], the Dementia Knowledge Twenty (DK-20) [11], and the Dementia Knowledge Assessment Tool (DKAT) [12]. Some had been developed with focuses on biomedical domains or lacking generalizability. Some have been outdated with poor psychometric vigor [13, 14]. To overcome some of the limitations of previous dementia knowledge tools, Annear and colleagues [15] developed the 27-item Dementia Knowledge Assessment Scale (DKAS) to measure dementia knowledge and reflect broader information about the condition related to dementia, and the items was further reduced from 27 to 25 to avoid redundancy [16]. The DKAS has been confirmed as a reliable and valid measure of dementia knowledge for diverse populations. This scale is appropriate for use by health professionals, students, family caregivers, and the general public [16].

The DKAS has also been translated into a number of other languages, such as Simplified Chinese [17], Japanese [18], and Mandarin and Bahasa Melayu for Singaporean [19], and it had been tested on hospital health care providers, allied health students and professionals, and families. However, the DKAS has not been translated into traditional Chinese and tested on long-term care staff, and it may need refinement and examination of the psychometric properties to assure its validity. The purpose of this study was to translate the 25-item DKAS from English to traditional Chinese and evaluate its psychometric properties among home care workers in Taiwan.

## Methods

### Study design and sample

A cross-sectional survey was conducted to test validity and reliability of the translated traditional Chinese version of the DKAS (DKAS-TC). This study was conducted over the period of March, 2019 to March, 2020. The inclusion criteria included: (1) aged between 20 and 65 years and able to provide consent; (2) being home care worker for at least 3 months and caring for clients with dementia; (3) able to read and write Chinese and fill in the questionnaire. A convenience sample of 285 home care workers who met inclusion criteria from three home care agencies in eastern Taiwan was invited and recruited to participate in the survey. A total of 252 participants completed the questionnaires, giving a response rate of 88.4%.

### Instrument

The DKAS is a 25-item scale to measure knowledge related to dementia. It consists of four subscales, including causes and characteristics (7 items), communication and behavior (6 items), care considerations (6 items), and risks and health promotion (6 items). Five response options were offered for each items: false, probably false, probably true, true, and I don’t know. The DKAS scoring system is as follows: two points for an answer of ‘true’ to a truthful statement or for an answer of ‘false’ to an untrue statement, one point for an answer of ‘probably true’ to a truthful statement or for an answer of ‘probably false’ to an untrue statement, zero point for an answer of ‘true’ or ‘probably true’ to an untrue statement, ‘false’ or ‘probably false’ to a truthful statement, or ‘I don’t know’. The total score was 50. The 25-item DKAS had good reliability with Cronbach’s alpha of .85 and acceptable subscale internal consistency with Cronbach’s alpha of .65–.76 [16].

### Translation procedures

Permission for using and translating the instrument was obtained from the original author. A forward translation and back translation process of the DKAS was referenced to a cross-cultural translation guidelines [20]. A translation team was formed with a coordinator, bilingual nursing scholars, translators, and a reviewer. Traditional Chinese is the official language in Taiwan. The original version of 25-item DKAS was translated from English into traditional Chinese by two bilingual Taiwanese nursing scholars who were proficient in English and Chinese and studied in an English speaking country for more than 10 years. A bilingual Taiwanese nursing professor familiar with dementia care then reviewed the first draft of the traditional Chinese translation to determine its relevance to Taiwanese situations both culturally and semantically. Next, back translation of the traditional Chinese version into English was carried out by two bilingual translators who were blinded to the original DKAS and had nursing work and translation experiences. The translation team then reviewed and reached a consensus on the wording of the traditional Chinese version. A few minor changes in wording were made, such as some verbs and adjectives. Cross-checking of the scale was to confirm that the translation did not result in any loss or alteration of meaning among translated scale items.

Pre-testing of the DKAS-TC was conducted in a group of ten home care workers with different levels of educational background through face-to-face interviews to make sure that the translated version was easy to understand for face validity. Home care workers were asked to provide suggestions to improve clarity. Minor modifications were made based on the feedback provided during the interviews to assure that the meaning of the items could be clearly understood. The final version was then developed by the final consensus of the translation team. The DKAS-TC required approximately 15 min to complete.

### Data collection

The study was approved by the Research Ethics Committee of Yuli hospital, Ministry of Health and Welfare in Taiwan (IRB No. YL-IRB- 10712). The researchers contacted with the administrators of three home care agencies in eastern Taiwan and asked for permission to conduct the study. The researchers explained the aim of the study and invited eligible potential home care workers to participate the study during their monthly meeting at the agencies, and those who signed the consent form were provided with a copy of a demographic questionnaire and the traditional Chinese version of the DKAS scale. The demographic questionnaire comprising age, gender, marital status, educational level, type of certification, workplace, and years of clinical experience was used to collect relevant data from the home care workers. The participants took about 15–20 min to complete the questionnaires. All participants were informed that participation was voluntary, and anonymity were preserved by de-identification and data aggregation.

### Data analysis

Statistical analyses were performed with IBM SPSS Statistics for Windows, Version 25.0.0.1 (Armonk, New York, USA). Descriptive statistics were computed using frequency and percentage or mean and standard deviation (SD), as appropriate. The internal consistency reliability of the DKAS-TC total scale and subscales were determined using Cronbach’s alpha coefficients and McDonald’s omega [21]. A Cronbach’s alpha and McDonald’s omega of .70 or higher was adopted as the criterion for representing an acceptable internal consistency [21–23].

Confirmatory data analysis (CFA) was conducted to examine the factor structure of the DKAS-TC using IBM SPSS Amos, Version 21.0 (Armonk, NY: IBM Corp.) and R (version 3.6.3, R Foundation for Statistical Computing, Vienna, Austria) with the lavaan package [24]. A diagonally weighted least squares (DWLS) was used as the estimation method for the Likert type ordinal data [25]. The goodness-of-fit of the CFA model was evaluated using normed chi-square divided by degrees of freedom (χ2/df), comparative fit index (CFI), Tucker-Lewis index (TLI), and the root mean square error of approximation (RMSEA). A χ2/df < 3, a CFI value > .90, a TLI value > .90, and a RMSEA < .08 indicate a good fit [26].

## Results

### Participant characteristics

The demographic characteristics of the sample are shown in Table 1. The majority of the participants (94%) were female, with a mean age of 46.87 years (SD = 8.19). 65.5% were being married. 66.3% had completed at least senior high or vocational school education. However, there were 33.7% who received education less then high school degree. The mean years of working as a home care workers were 7.49 years (SD = 5.30). Only 26.6% had received on-the-job dementia care training more than 12 h in the past year, and 18.7% had not received any dementia training courses.

**Table 1:** Demographic characteristics of participants (*N* = 252)

Variable	n	Percentage (%)
Sex		
male	15	6.0
female	237	94.0
Marital status		
being married	165	65.5
divorce	40	15.9
widowed	30	11.9
unmarried	17	6.7
Education		
elementary school degree	27	10.7
junior high school degree	58	23.0
senior high school and vocational school degree	167	66.3
On-the-job dementia care training in the past year, hours		
None	47	18.7
< 2	43	17.1
3–6	44	17.5
7–12	51	20.2
> 12	67	26.6
Variable	Mean	SD
Age (years)	46.87	8.19
Clinical experience as home care workers (years)	7.49	5.30

*Note*: *SD* standard deviation

### Confirmatory factor analysis results

**Fig. 1:**
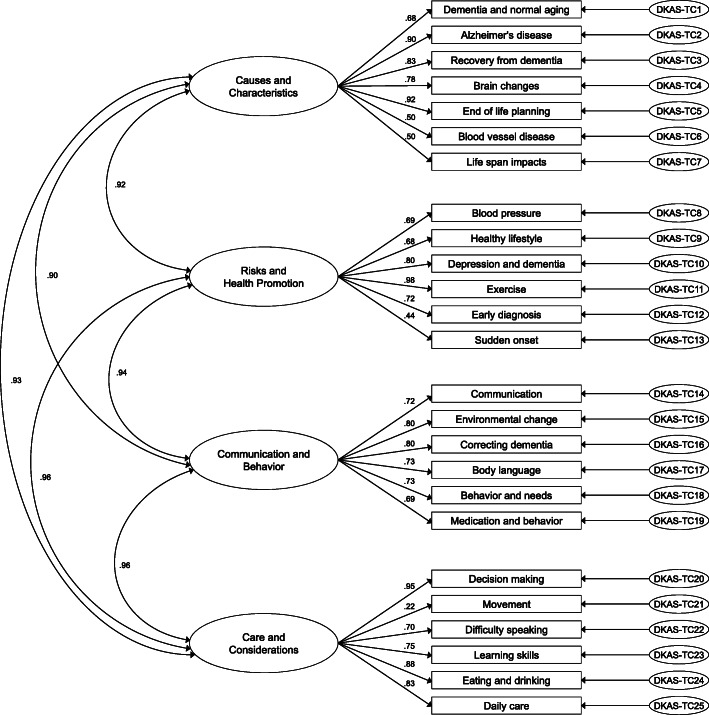
Confirmatory factor analysis of the four-factor model of the DKAS-TC

The four-factor model of DKAS-TC was demonstrated by the indices of CFA which performed moderately well with a population of Taiwanese home care workers. All items, except item 21 (“movement is generally affected in the later stages of dementia”) within each factor had acceptable factor loadings above .4. The 25-item, four-factor model exhibited adequate model fit (χ2/df = 0.642, CFI = .999, TLI = .998, RMSEA = .012). The item numbers in each of four domains were: “causes and characteristics “(7 items), “risks and health promotion” (6 items), “communication and behavior” (6 items), and “care considerations” (6 items). The accepted CFA model is presented in Fig. 1.

The CFA results for the 25-item DKAS-TC supported the four-factor structure of the original English version. Each item contributed to its expected subscale, indicating evidence of construct validity. Our esult is similar with the results of the original 25-item English version [16], 25-item simplified Chinese version in China [17], and the 18-item Japanese version [18].

### Internal consistency reliability and homogeneity

The DKAS-TC achieved a good overall Cronbach’s alpha of .93 and McDonald’s omega of 0.94. The internal consistency reliabilities for the four domains were as follows: “causes and characteristics” (Cronbach’s alpha = .81; McDonald’s omega = .82), “risks and health promotion” (Cronbach’s alpha = .76; McDonald’s omega = .77), “communication and behavior” (Cronbach’s alpha = .80; McDonald’s omega = .80), and “care considerations” (Cronbach’s alpha = .80; McDonald’s omega = .82).

The Pearson’s correlation coefficients between four factors ranged from .71 to .79 (*p* < .001) with high degree of positive correlations between factors, indicating sufficient independence among the subscales. No exceptionally high correlations were noted. The results demonstrated good reliability and homogeneity between factors and the DKAS-TC scale (Table 2).

**Table 2:** Correlations between four factors of the DKAS-TC

Factor	Causes and characteristics	Communication and behavior	Care considerations	Risks and health promotion
Causes and characteristics	1			
Communication and behavior	.74	1		
Care considerations	.71	.76	1	
Risks and health promotion	.71	.76	.73	1

All Pearson’s correlation coefficients are significant at *p* < .001

### Dementia knowledge

The mean score for the 25-item DKAS-TC was 25.68 (SD = 13.73) out of the total score of 50 among Taiwanese home care workers. The mean scores of the four subscales ranged from 5.33 to 7.63, with the lowest mean score (mean = 5.33, SD = 3.50) for “risks and health promotion” subscale and the highest mean score (mean = 7.63, SD = 3.58) for “care considerations” subscale. Higher score on a factor indicates a greater understanding towards the particular aspect of dementia knowledge (Table 3).

**Table 3:** Mean scores for four factors of the DKAS-TC

Factor	Maximum total	Mean	SD	Range
Causes and characteristics	14	6.54	4.34	0–14
Risks and health promotion	12	5.53	3.50	0–12
Communication and behavior	12	5.99	3.93	0–12
Care considerations	12	7.63	3.58	1–12
DKAS-TC total score	50	25.68	13.73	2–48

## Discussion

We translated the DKAS from English to traditional Chinese and examined the psychometric properties of the DKAS-TC in a sample of Taiwanese home care workers. The DKAS-TC had cultural modifications in wording during translation process. The DKAS-TC shows good reliability and validity, indicating that it can be used to measure dementia knowledge level among home care workers. In our study, a rigorous process was followed for instrument translation.

The 25-item of DKAS-TC had good reliability for the total scale and the subscales as shown by the Cronbach’s alpha and McDonald’s omega of .70 or higher. Our results were comparable to those of the original 25-item English version [16], the simplified Chinese version [17], and the Japanese version [18].

CFA was used to examine the psychometric properties of the DKAS-TC. The CFA results for the 25-item DKAS-TC supported the four-factor structure of the original English version. Each item contributed to its expected subscale, indicating evidence of construct validity. This result is similar with those of the original 25-item English version with the sample of international cohort health professionals [16], 25-item simplified Chinese version in China [17], and the 18-item Japanese version [18]. However, the 23-item version with the sample of Singapore informal caregivers found three-factor model [19]. The differences of factor structure found in the studies might be due to differences in the sample. The majority of the samples in previous studies were nurses [15], health students and professionals in Japan [18], an international cohort of health professionals [16], and hospital and nursing home health care providers in China [17] except only one study from Singapore in which the sample was informal caregivers [19]. Our study specifically recruited only home care workers in Taiwan.

Our study result revealed that home care workers had moderate level of dementia knowledge. The mean score of the DKAS-TC in our study was 25.7 out of 50, indicating moderate level. The dementia knowledge of home care workers in our study was lower than those of the previous studies with samples of health professionals. A study found that the mean scores of DKAS in the international cohort of health professionals ranged from 32.5 to 37.1 out of 50, indicating above moderate levels [16]. Health professionals in hospitals and care homes in China [17] also had higher dementia knowledge (mean score of 29.8 out of 50) compared to the home care workers in our study. The study of Japanese sample of health students and health professionals also had above moderate level of dementia knowledge (mean score of 20.7 out of 36) [18]. Another study [27] found a sample of Australian nurses, allied health professionals, care workers, and care managers had much higher dementia knowledge (mean score of 44.9 out of 54) compared to that of our study. However, our study result was similar with that of a study in which the sample of informal caregivers in Singapore had only moderate level of dementia knowledge with a mean score was 24.1 out of 46 [19].

At the item level, our study found that home care workers had lowest mean scores on two items: “blood vessel disease (vascular dementia) is the most common form of dementia” and “the sudden onset of cognitive problems is characteristic of common forms of dementia”. This result was similar to those of previous studies [17, 18, 27]. Our result also indicated that home care workers had less knowledge on the subscales of “risks and health promotion” and “communication and behavior” compared to the other two subscales.

The levels of dementia knowledge may be varied due to cultural, education level, and sociodemographic differences [28] The inadequate dementia knowledge level of home care workers in our study could be the result of a lower level of education and limited dementia care training. In Taiwan, the majority of home care workers had an education level of senior high school and vocational school degree or lower, which were lower than those in previous studies. More than 30% of home care workers had only an educational level of less then high school degree in our study. In addition, our study also showed that the majority of the home care workers did not receive adequate training on dementia care. Approximately 75% of participants had received on-the-job dementia care training less than 12 h in the past year. In addition, high turnover rate in Taiwan may prevent home care workers from receiving adequate on-the-job dementia training in the institutions. In our study, one fourth of the home care workers had only worked as home care workers at current institutions less than 1 year, indicating this group might not have opportunities to receive adequate dementia training with less experiences in caring for those with dementia. Our study revealed the need to provide home care workers in Taiwan more dementia care training to improve their dementia knowledge. More dementia education or training for health professionals [16–18] and informal caregivers [19] were also suggested by previous studies. Therefore, the content and training strategies for dementia care should be tailored to the needs of different groups who involve with care for those with dementia [17, 19, 27].

### Limitations

This study had several limitations. Firstly, our study recruited home care workers only in rural areas of eastern Taiwan, and therefore our findings might not be generalizable to home care workers in metropolitan area and other health care professionals in Taiwan. Secondly, females are the major workforce in the home care service in Taiwan. In our study, males only made up 6% of our participants. This characteristic of home care workforce is typical in many Asian countries. However, our study results might not be generalizable to countries with a high proportion of males in the home care workforce. Thirdly, the concurrent validity was not evaluated in this study. The Chinese version of the 30-item Alzheimer’s Disease Knowledge Scale (ADKS) [29] can be used for assessing the concurrent validity of our traditional Chinese translated version of DKAS in future study. Finally, test-retest reliability of the DKAS-TC was not performed in this study. Future research is recommended to reconfirm the validity and reliability of the DKAS-TC with other health professionals in Taiwan or countries where traditional Chinese is official language.

## Conclusions

To our knowledge, this is the first study that examined the psychometric properties of the DKAS-TC in a sample of home care workers in Taiwan. The study findings confirmed that the 25-item four-factor DKAS-TC had adequate construct validity and internal consistency among Taiwan home care workers. DKAS-TC provides a useful tool to assess dementia knowledge, identify knowledge deficit, and can be used to evaluate the effects of dementia training or education. Our study findings also indicated that the level of dementia knowledge among home care workers in Taiwan was inadequate. Given the increasing care demands of people with dementia in Taiwan and home care workers as the major frontline home care workforce, there is a need for developing suitable dementia care training tailored to their learning needs and educational levels, and to improve their quality of care for those with dementia in Taiwan.

## Data Availability

The datasets used and/or analyzed during the current study are available from the corresponding author on reasonable request.

## Abbreviations

DKAS-TC: Dementia Knowledge Assessment Scale-Traditional Chinese
CFA: Confirmatory Factor Analysis
ADKS: Alzheimer’s Disease Knowledge Scale
DK-20: Dementia Knowledge Twenty
DKAT: Dementia Knowledge Assessment Tool
DWLS: Diagonally Weighted Least Squares
CFI: Comparative Fit Index
TLI: Tucker-Lewis Index
RMSEA: Root Mean Square Error of Approximation
